# Alginate Formulations: Current Developments in the Race for Hydrogel-Based Cardiac Regeneration

**DOI:** 10.3389/fbioe.2020.00414

**Published:** 2020-05-08

**Authors:** Giada Cattelan, Amparo Guerrero Gerbolés, Ruben Foresti, Peter P. Pramstaller, Alessandra Rossini, Michele Miragoli, Cristina Caffarra Malvezzi

**Affiliations:** ^1^Institute for Biomedicine, Eurac Research, Affiliated Institute of the University of Lübeck, Bolzano, Italy; ^2^Department of Medicine and Surgery, University of Parma, Parma, Italy; ^3^CERT, Center of Excellence for Toxicological Research, University of Parma, Parma, Italy

**Keywords:** alginate, hydrogels, biomaterials, heart failure, cardiac regeneration, clinical trials

## Abstract

Cardiovascular diseases, including myocardial infarction (MI), represent the main worldwide cause of mortality and morbidity. In this scenario, to contrast the irreversible damages following MI, cardiac regeneration has emerged as a novel and promising solution for *in situ* cellular regeneration, preserving cell behavior and tissue cytoarchitecture. Among the huge variety of natural, synthetic, and hybrid compounds used for tissue regeneration, alginate emerged as a good candidate for cellular preservation and delivery, becoming one of the first biomaterial tested in pre-clinical research and clinical trials concerning cardiovascular diseases. Although promising results have been obtained, recellularization and revascularization of the infarcted area present still major limitations. Therefore, the demand is rising for alginate functionalization and its combination with molecules, factors, and drugs capable to boost the regenerative potential of the cardiac tissue. The focus of this review is to elucidate the promising properties of alginate and to highlight its benefits in clinical trials in relation to cardiac regeneration. The definition of hydrogels, the alginate characteristics, and recent biomedical applications are herewith described. Afterward, the review examines in depth the ongoing developments to refine the material relevance in cardiac recovery and regeneration after MI and presents current clinical trials based on alginate.

## Introduction

The incidence of cardiovascular diseases (CVDs) accounts for most of non-communicable disease (NCD) deaths annually^1^ and has increased over the last few decades, primarily because of the expansion of the “modern” lifestyle in wealthy countries and the lengthening of life expectancy. However, despite improvements in its management, CVDs still remain the major cause of death worldwide claiming 17.9 million lives a year, with an estimated 31% of all deaths globally.

Among CVDs, myocardial infarction (MI) remains one of the leading cause of heart failure, which, in turns, represents the commonest driver for mortality and morbidity (Cahill and Kharbanda, 2017). At present, there is no definitive treatment for MI, although great effort has been made to find new therapies and permanent solutions (Mitter and Yancy, 2017). The available treatments for MI and consequent heart failure are drugs, medical electronic devices and, in the most severe cases, heart transplants (Ferrini et al., 2019). One of the main problems about infarcted heart is cardiomyocytes incapability of self-regeneration along with lack of treatments able to restore the lost parenchyma, thus the impossibility to reestablish normal pump function. Regenerative medicine, combining tissue engineering and drug delivery, aims to repair damaged or diseased tissues and organs, and plays an important role in restoring normal condition in the infarcted heart. Some evidence showed that once applied onto MI, three dimensional printed cardiac patches, and cardiac-tissue derived extracellular matrix (ECM) scaffolds seem to have the potential to improve cardiac functions, cardiac support, and tissue regeneration, improving angiogenesis and reducing the scar size (Kc et al., 2019; Yeung et al., 2019). The regenerative medicine approach can be cell-free (Domenech et al., 2016; McLaughlin et al., 2019), or cell-based, comprising stem cell therapy *via* injection or grafting into the infarcted area, e.g., resident stem cells (Forrester et al., 2003; Leor et al., 2007), myoblasts (Sekiya et al., 2009) or by reprogramming fibroblasts directly into cardiomyocytes (CMs) (Ieda et al., 2010). However, there are several intrinsic limitations in restoring a complete and functional heart: (i) cardiomyogenesis is very low and incapable to repair the damaged areas (Zacchigna and Giacca, 2014; Leitolis et al., 2019), and the infarcted area displays a hostile environment for cells due to high level of oxidative stress, poor nutrient supply, and it offers an open way for host immune system attack; (ii) the recovery of the cardiac tissue requires maturation and completeness in mechanical, electrical, and chemical function (Pena et al., 2018); (iii) the majority of stem cells used for therapeutic purposes in amending the infarcted heart are lost within 1 day after the injection without using any vehicle-matrix; (iv) the balance of cell-removal, ECM preservation, and recellularization is challenging due to the residual presence of detergent and endotoxin in the patch (Kc et al., 2019). Therefore it emerges the necessity of looking for appropriate biomaterials in a suitable form as cell preservation and delivery system (van den Akker et al., 2017; Weinberger et al., 2017; Zuppinger, 2019). Biomaterials should provide biological, mechanical, electrical, and chemical support for the myocardium and they should mimic its physiological and homeostatic conditions (Pena et al., 2018). Nowadays, the biomaterials that comprise cell-types and scaffold properties are evolving (Foresti et al., 2019), as well as the application strategies for these 3D artificial cardiac tissues. Recently, thanks to novel hydrogel formulations and 3D printing technology, it was possible to create *ad hoc* cardiac patches (scaffold) for myocardial repair (Chachques et al., 2020), drug or nanoparticles delivery (Bheri and Davis, 2019; Foresti et al., 2020b) or injectable hydrogel for mechanical stabilization of the repaired area (Seif-Naraghi et al., 2014; Kambe and Yamaoka, 2019).

Hydrogels are a network of polymers of different origin: natural, synthetic, or hybrid (a mix of natural and synthetic) (Ye et al., 2011), extensively described and detailed classified by Saludas et al. (2017) and Pena et al. (2018). Hydrogels have the capability to absorb water, maintain their shape, and dissolve in the body according to controlled kinetics Gauvin et al., 2012; Saludas et al., 2017). One of the principal qualities of the hydrogel is to be able to pass through a tiny syringe needle (27–30 G), turning from liquid state at room temperature to gel-state in the body at 37°C (Sepantafar et al., 2016; Ferrini et al., 2019; Milazzo et al., 2019).

Of note, hydrogels showed promising results in cardiac regeneration in both preclinical models (Saludas et al., 2017) and in some clinical trials as mechanical support in the infarcted heart (Tous et al., 2011). Hydrogels give a significant contribution to heart tissue regeneration being capable of simulating a temporary artificial ECM (Saygili et al., 2019) supporting cell infiltration, adhesion, proliferation, and differentiation (Hasan et al., 2015). The most widely used hydrogel (Lee and Mooney, 2012; Sun and Tan, 2013) is the natural biomaterial alginate, which, together with decellularized ECM [already extensively reviewed in Spinali’s work, (Spinali and Schmuck, 2018)], represents the most promising material in cardiac regeneration. Degradable and bioactive microstructures of alginate-based hydrogel showed a great promise on the recovery of left ventricular function as well as the release of cardioactive substances (Hynes, 2009) in the target tissue.

## Alginate

Sodium alginate is a natural polymer found in brown algae cell walls, including *Macrocystis pyrifera*, *Laminaria hyperborea*, *Ascophyllum nodosum* (Sachan et al., 2009) and in several bacteria strains (*Azotobacter*, *Pseudomonas*) (Remminghorst and Rehm, 2006). Alginate’s hydrogels formed through physical crosslinking present highly tunable mechanical properties (Hecht and Srebnik, 2016), and thanks to its versatile and biological properties alginate is widely used in biomedical research Cheng et al., 2012; Ruvinov and Cohen, 2016; Saludas et al., 2017; Dhamecha et al., 2019). In this section, we intend to provide a brief insight into alginate characteristics.

### Chemical Properties

Alginate is an anionic polysaccharide. It is a linear copolymer consisting of random sequences of 1,4-linked β-D-mannuronic acid (M) and 1,4 α-L-guluronic acid (G) residues (Hecht and Srebnik, 2016). Commercial alginate presents different ratios of M and G residues and block-length depending on its source; consequently, molecular weight (expressed as an average of all the molecules in the sample) is variable and changes in a range between 33,000 and 400,000 g/mol. Regarding polymer’s solubility, it is important to underline that whereas alginic acid is insoluble in water and organic solvents, alginate monovalent salts and esters are water-soluble and form stable viscous solutions (Szekalska et al., 2016).

### Physical Properties

Alginate physical properties strictly depend on its composition and concentration, which makes its characterization difficult, but allows for a wide range of customization according to the desired application (implantation, ECM mimic, etc.). An increase of polymer concentration in the solution coincides with an increase of gel stiffness, particularly when using high-molecular weight alginates that maintain long-range interactions within the gel (LeRoux et al., 1999). Nevertheless, increasing polymer concentration also implies an increased viscosity of the pre-gelled solution, which makes this strategy less feasible when cells or active ingredients need to be included (Kong et al., 2003). In order to modulate the stiffness without altering the viscosity, a possible strategy is the combination of high and low-MW alginates in specific ratios (Kong et al., 2002) or alternatively, the formation of polyelectrolyte complexes through the addition of cationic poly-(ethyleneimine) that increases the resistance to de-crosslinking (Kong and Mooney, 2003).

Another relevant criterion that determines alginate physical behavior is the crosslinking reaction. Many methods have been described for alginate crosslinking, including ionic crosslinking, covalent crosslinking, phase transition (thermal gelation), “click” reaction, free radical polymerization (Sun and Tan, 2013) and lowering the pH value below the pKa of alginate monomers using lactones, such as d-glucono-δ-lactone (Sachan et al., 2009). However, among these strategies, the most widespread is ionic crosslinking since allows instantaneous and almost temperature-independent solution/gel transition in relatively mild conditions, in presence of multivalent cations, physical crosslinking is instantaneous and almost temperature-independent.

Moreover, several studies revealed that, in presence of divalent ions, alginate solution/gel transition occurred under physiological conditions, such as the acidic environment of the body fluids (Fu et al., 2011). In particular, many of the technological applications of alginate rely on the gelation in presence of Ca^2+^ in a two-step process that leads to the formation of inter- and intra-chain bridging. Calcium ions interact with the acidic sites on the G residues leading to chain-chain association; following, the tightly linked dimers form weak interdimer associations mainly governed by electrostatic interactions among the dimers with higher Ca^2+^ concentration. This ion-chain interaction involves the formation of a cage-shape association of chain regions rich in G, implying that poly M-blocks and alternating MG blocks display lower selectivity toward Ca^2+^ (Fu et al., 2011; Hecht and Srebnik, 2016). Therefore, gelling properties depend not only on alginate molecular weight, but are also strongly associated with its structure and composition in terms of M-, G-, and MG-blocks (Sachan et al., 2009). High G-blocks content determines low shrinkage during gel formation and renders alginate stiffer, mechanically stable and more permeable due to larger pores’ size (Martinsen et al., 1989), while high M-blocks content are required when alginate needs additional coating, such as enrichment of RGD sites to mediate cell adhesion (Kuo and Ma, 2001; Niekraszewicz and Niekraszewicz, 2009; Fu et al., 2011). Prevalence of MG-blocks, instead, give rise to alginate shrinkage and higher flexibility (Jorgensen et al., 2007; Szekalska et al., 2016).

Another relevant aspect to consider in the gelation process is the velocity of the reaction. Slow gelation provides uniform and ordered gel network structures with increased tensile properties and mechanical integrity (Kuo and Ma, 2001; Drury et al., 2004). One of the strategies to reduce the rate of gel forming process is the application of phosphate buffers (e.g., sodium hexametaphosphate); since the affinity of phosphate for calcium is higher compare to that of alginate, the presence of phosphate ions exert a chelating action and delay alginate gelation process (Dainty et al., 1986; Xu et al., 2007) alternatively, is possible to use calcium sulfate and calcium carbonate as Ca^2+^ sources: the lower solubility of these reagents prolongs gel formation. Lastly, another approach concerns the regulation of temperature: at lower temperatures, the reactivity of Ca^2+^ is reduced and allows the control of hydrogel formation (Augst et al., 2006; Zhao Y. et al., 2016).

#### Mechanical Properties

Alginate-based hydrogel mechanical properties strictly depend on the number and concentration of M and G residues (Augst et al., 2006). If G overcomes M, the hydrogel displays high mechanical stiffness. Therefore, by varying G and M content it is possible to change the elastic modulus. It was observed that elastic modulus homogeneity among 3D printing alginate-based scaffolds strictly depends on the gelation rate (Mahdi et al., 2016); slow processes result in greater homogenous scaffold (Kuo and Ma, 2001). Kaklamani et al. (2014) demonstrated how the gelation process can be modulated using divalent cations (Mg^2+^, Ca^2+^, and Sr^2+^), resulting in a tight controlled Young modulus of elasticity as a function of cations and alginate concentration.

Recently, it was demonstrated that xylitol, mannitol, or peptides can modify the gelation process increasing strength and elasticity (Ochbaum et al., 2018). The swelling properties of alginate are also modulators of mechanical strength. Swelling degree is inversely dependent on gelation time (bt Ibrahim et al., 2019). Sodium alginate films immersed in CaCl_2_ solution for 2 min denote a swelling degree of 51% compared to those immersed for 8 min (26%), suggesting that Ca^2+^ ions can penetrate into the film matrix and interact with sodium ions improving scaffold integrity and final geometry. In particular, in order to increase the volume of a given bio-based device it is necessary to increase the “surface-to-volume ratio” and engineering the structure (Elviri et al., 2017; Foresti et al., 2019) by jellifying every single deposed layer (Campisi et al., 2018).

Finally, it becomes imperative to characterize the alginate mechanical properties in light with the method of fabrication (simple deposition/injection or scaffold manufacturing via 3D printer) (Ozbolat and Hospodiuk, 2016) and for the final use as a biomaterial (Ravnic et al., 2017).

### Biological Properties

Alginate is regarded as biocompatible, non-immunogenic, and non-toxic material (Sachan et al., 2009). Alginate cross-linked gels are not degradable in mammalian digestive tract but the elution of the multivalent ions eventually leads to its dissolution; moreover, a physiological concentration of sodium ions has been proved to alter alginate’s shear properties inducing a “softening effect” on the hydrogel’s matrix (LeRoux et al., 1999). Alginate biocompatibility was confirmed *in vivo* after ocular (Lin et al., 2004), topical (Coskun et al., 2014), local (Veriter et al., 2010; Chang et al., 2012), and oral administration (Sosnik, 2014). Moreover, Food and Drug Administration has categorized several alginate salts (calcium, sodium, ammonium, and potassium) as well as propylene glycol alginate derivative as generally regarded as safe (GRAS) ingredients for oral administration^2^ (Szekalska et al., 2016).

Besides being considered a safe polymer, alginate also presents some properties that enhance its attractiveness for biomedical applications, such as bio-adhesivity and antibacterial/-viral activity.

The presence of free carboxyl groups provides good mucoadhesive properties to alginate and allows its interaction with mucin by hydrogen and electrostatic bonding (Hecht and Srebnik, 2016; Szekalska et al., 2016). This property is strongly influenced by environmental factors such as pH, since only ionized carboxyl groups can interact with mucosal tissue. Moreover, different soluble formulations of alginate facilitate solvent penetration through its matrix, resulting in more viscous and cohesive gel structures that strengthen the mucoadhesive bonds; yet, an excessive hydration in physiological fluids might weaken mucoadhesiveness as a results of attenuation of the functional groups available for interactions (Taylor et al., 2005; Mythri et al., 2011; Haugstad et al., 2015). Ionized carboxyl groups also determine the reported antibacterial activity against a wide variety of species, including Pseudomonas, Escherichia, Proteus, and Acinetobacter (Khan et al., 2012; Pritchard et al., 2016), negatively charged alginate interacts with the outer bacterial cellular surface leading to its disruption (Yan et al., 2011; Benavides et al., 2012). Furthermore, the formation of a viscous layer around the bacterial cell prevents nutrient transport, decreases membrane function (Yan et al., 2011), and can exert chelation processes responsible for modulating the production of toxins, microbial growth and other crucial factors for microorganisms stability (Szekalska et al., 2016). Alginate antiviral activity relies on sulfated polysaccharides and alginic acid-containing fractions extracted from algae. The mechanism of action may be related to the strong anionic charge of sulfated alginate, capable of interacting with the positively charged host cell and, as a result, preventing virus contact with the host cell (Witvrouw and De Clercq, 1997; Sano, 1999; Meiyu et al., 2003). At present, antiviral efficacy has been reported against Flaviviridae, Togaviridae, Rhabdoviridae, and Herpesviridae viruses’ families (Son et al., 2003; Lee et al., 2011; Wang et al., 2014; Ahmadi et al., 2015). Other relevant properties concern the modulation of several responses to pathology, from immunostimulation to anti-oxidant and anti-inflammatory activities. It has been reported that alginate with high M-block content is able to activate macrophages and monocytes leading to the secretion of cytokines and cytotoxic factors (Son et al., 2001). The anti-inflammatory cytokines secreted by monocytes, eventually lead to attenuate the production of nitric oxide, reactive oxygen species (ROS), prostaglandin E2, and cyclooxygenase COX-2, determining therefore alginate anti-oxidant effect (Rocha de Souza et al., 2007; Yamamoto et al., 2007; Maciel et al., 2013; Namvar et al., 2013). Calcium alginate displays hemostatic efficacy through platelets activation and thrombin generation (Hattori et al., 2010), and it has been reported to lower blood pressure as a results of calcium antagonist activity, especially toward voltage-operated calcium channels (Chaki et al., 2007; Terakado et al., 2012).

### Alginate in Biomedical Research

Alginate due to its versatile and biologically properties such as biocompatibility, (possible) non-immunogenicity, chelating ability, water solubility, flexibility (it can be easily modified in any form) and low-cost, is widely used in biomedical research (Sun and Tan, 2013). In particular, alginate is used in protein/drug delivery systems, tissue regeneration, and wound healing (Miao et al., 2018). To date, there are several preclinical and clinical studies (see paragraph below) using alginate as a cargo system to control delivery bioactive agents, e.g., growth factors (Pawar and Edgar, 2012), cytokines (Lee and Mooney, 2012), doxorubicin (Paques et al., 2014), paclitaxel (Wu et al., 2014). Moreover, alginate is also used as an excipient in several drugs due to its gel forming, stabilizing and thickening properties, e.g., Gaviscon^®^, Bisodol^®^, Asilone (Miao et al., 2018). Regarding tissue regeneration, unless big steps have been done, there is still a considerable gap between research and clinical application of alginate in tissue regeneration. More studies should be performed in terms of improving alginate characterization, functionalization, biodegradability, and mechanical properties in order to facilitate tissue and organ regeneration. To date, several studies have been done implying use of alginate in cardiac regeneration (Rodness et al., 2016; Foresti et al., 2020a), skin regeneration (Yang et al., 2019), osteo and cartilage regeneration (Venkatesan et al., 2015; Calasans-Maia et al., 2019; Yang et al., 2019), and neural tissue regeneration (Homaeigohar et al., 2019). Due to its hydrophilicity, capability to adsorb wound exudate and maintaining a moist microenvironment alginate is suitable for wound dressing. Alginate dressings can be prepared by ionic crosslinking (with calcium, magnesium, etc.) to form a gel, or followed by processing to form freeze-dried porous sheets in form of foams, and fibrous dressings (Aderibigbe and Buyana, 2018). At present, there are several alginate dressings commercially available including Algicell^TM^, Fibracol^TM^Plus, Hyalogran^®^, and Tromboguard^®^.

## Functionalized Alginate

**FIGURE 1:**
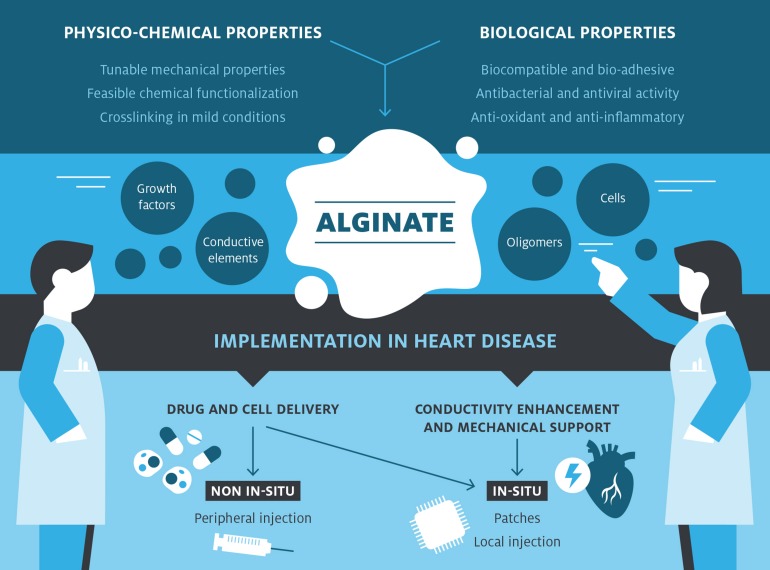
Alginate-based devices development in cardiac treatment. Alginate displays some physico-chemical and biological advantageous characteristics that, combined together, render this biomaterial suitable for further modification. Possible modifications and implementations are: the inclusion of different cell types, bioactive molecules, functional oligomers, and conductive materials to provide further functionalities to the pristine material addressing the main issues in cardiac regeneration field. Thanks to its functionalization, alginate can be implied either as a delivery system or as a support material in cardiac regeneration choosing the most efficient way of administration, *in situ* or not.

As mentioned in the introduction, the heart is a complex electromechanical organ. After MI, it occurs loss of CMs, lack of angiogenesis, and conductive connection disruption that brings to a malfunctioned heart. Lack of treatments to restore lost cardiomyocytes and cardiomyocytes incapability of self-regeneration brought scientists to think about regenerative medicine as a winner strategy to restore normal functionality of the infarcted heart. Alginate emerged as a promising natural polymer in cardiac regeneration, supporting heart vascularization, re-cellularization, and restoring electrical conductivity (Figure 1). In this section, it will be provided an overview of alginate’s roles in restoring normal functionality of infarcted heart; the studies took into consideration are summarized in Table 1.

**TABLE 1:** Promising alginate-based systems in cardiac regeneration.

**Material**	**Factors**	**Cell type**	**Model**	**Administration**	**Treatment timing**	**Monitoring time**	**Results**	**References**
Alginate, gelatin	VEGF	rMSCs	Sprague-Dawley rats, left coronary artery block and reperfusion after 45′	VEGF-encapsuled rMSCs injection via tail vein	2 days after myocardial injury	6 weeks	Increased vascular density and perfusion rate in the border zone; restored indexes of ejection fraction and fractional shortening.	Liu et al., 2017
Alginate, gelatin		HUVECs	Immunocompromised Balb/c mice	Microcapsules injection in quadriceps muscle	3 days after cyclophosphamide injection	1 week	Increase of vascular density; endothelial cell alignment; peripheral epithelization.	Nemati et al., 2017
Alginate-chitosan PEC		MSCs	Lewis rats	Implantation of a macroporous scaffold in the pectoralis major muscle	12 h after scaffold hydration	1–4 weeks	Good engraftment and gradual scaffold degradation; functional blood vessels formation; reduction of the fibrous capsule.	Bushkalova et al., 2019
Alginate-chitosan micromatrix		mPSCs pre-differentiated into early cardiac lineage	C57BL6/J mice, permanent LAD ligation	Three injections in the periphery of infarcted tissue	5 min after myocardial injury	4 weeks	Increased cell retention, survival and migration into MI zone; improved ejection fraction, stroke volume, cardiac output and left ventricle end-systolic and end-diastolic volumes; reduced fibrosis ad hypertrophy.	Zhao S. et al., 2016
Alginate, chitosan	VEGF		Sprague-Dawley rats, permanent LAD ligation	Microspheres reinforced with chitosan net and applied on the LV epicardial surface	4 days after myocardial injury	4 weeks	Better fractional shortening; higher cell infiltration and vascular-like structures; increased scar thickness and decreased scar area and length.	Rodness et al., 2016
2-aminopyridine-5-thiocarboxamide, alginate-CHO, tetraaniline, gelatin		ADSCs	Sprague-Dawley rats, LAD ligation	Hydrogel injections adjacent to MI area	Immediately after myocardial injury	1–4 weeks	Good electroactivity *in vitro*; increased cell retention *in vivo*; improved ejection fraction; decreased fibrotic area and wall thinning; increased expression of VEGF and Ang-1.	Liang et al., 2019
Alginate, gold nanowires			Neonatal rat cardiomyocytes and fibroblasts	Cells were seeded onto the nanocomposite scaffold, and cultured for 3 days		3–8 days	Increased cell alignment; higher levels of proteins involved in contraction and electrical coupling; synchronous contraction and calcium transients in temporal sequence.	Dvir et al., 2011

### Alginate in Support of Heart Vascularization

A crucial hurdle in the recovery post MI is lack of angiogenesis that leads to the irreversible loss of cardiomyocytes in the first 2–4 h of the onset (Thygesen et al., 2007), thus contributing to the progression of maladaptive ventricular remodeling, including scar formation and ventricular dilatation, that can eventually lead to heart failure (French and Kramer, 2007). To address this deficiency in neovascularization both delivery of factors and/or cells have been explored. Specifically, most works are focused on delivering of mesenchymal stem cells (MSCs) and vascular endothelial growth factor (VEGF). Beneficial effects of MSCs on MI were already reported in literature (Gnecchi et al., 2008); their impact was mainly related to an hypoxia-dependent secretion of several cytokines, chemokines, and growth factors, that not only exert a cytoprotective effect on cardiomyocytes survival (Gnecchi et al., 2005), but lead also to an increase in collateral perfusion through paracrine stimulation of proliferation and migration of endothelial cells (ECs) and vascular smooth muscle cells (VSMCs) (Kinnaird et al., 2004). Marrow-derived stromal cells express genes encoding a broad spectrum of arteriogenic cytokines and promote *in vitro* and *in vivo* arteriogenesis through paracrine mechanisms (Nagaya et al., 2004). VEGF, instead, is the most powerful and well-characterized proangiogenic cytokine (Maharaj and D’Amore, 2007) associated with improvements in cardiac vascularization (Oduk et al., 2018). Unfortunately, delivery and retention of cells or growth factors to the damaged area remains critical. Moreover, VEGF delivering may lead to severe side effects, such as hypotension, limb edema, and retinopathy, growth of tumors and/or metastasis (Zietz et al., 1998; Baumgartner et al., 2000; Gasparini, 2000; Lee et al., 2000; Freedman and Isner, 2002; Henry et al., 2003). Given these considerations, it becomes clear why so much effort is put into defining an appropriate cell or molecules delivery system in order to help vascularization in the infarcted heart. In this scenario, several studies demonstrated alginate suitability as support material. Rodness et al. (2016) developed a VEGF-loaded patch made of compacted alginate microspheres, retained by a chitosan sheet. In order to combine alginate with the growth factor, the authors mixed recombinant VEGF with Na^+^-alginate solution and produced microspheres using a water-in-oil-in-water double emulsion technique with pure olive oil and ddH_2_O, and CaCl_2_ crosslinking; microspheres were following compacted by centrifugation. From a certain point of view, this strategy seems to recall and overcome the approach of current clinical trials based on the mere injection of alginate in the ventricular wall. The rationale behind this approach relies on the highly tunable degradation of microspheres, that allows the controlled release of pro-angiogenic factors, and the restraint properties of a condensed microsphere patch reinforced with chitosan. After assessing the biological activity of VEGF-loaded patches *in vitro*, they were implanted on the epicardial surface of a MI-rat model. Both VEGF-loaded and control patches displayed similar functional outcomes in terms of fractional shortening, cell infiltration, wall thickness, scar area and length, to indicate that microsphere patch and chitosan sheet system may function as a ventricular restraint device that limits the extent of scar expansion post-MI. In addition, the team reported that VEGF-loaded patches maintained their biological activity *in vivo* up to 4 weeks after implantation, enhancing the spontaneous angiogenesis and vasculogenesis within the compacted alginate microspheres. An alternative approach to deliver VEGF to the infarcted area was proposed by Liu et al. (2017), who investigated the potential of MSCs as carriers of pro-angiogenetic factors. The team took advantage of MSCs natural tropism to the infarcted area (Lau and Wang, 2011) to deliver VEGF. They performed a layer-by-layer (LbL) self-assembly coating where alginate provided cohesion between two layers of gelatin embedded with VEGF. This tiny-film fabrication technique consisted in alternating steps of coating and washing, in which cells were progressively incubated with gelatin, alginate and gelatin. This strategy guarantees the maintenance of the metabolic activity of the encapsulated material and endows protection (Yang et al., 2011) without affecting cell viability, proliferation and differentiation (Li et al., 2015). Other interesting approaches to enhance vascularization involving cell delivery were successfully tested on muscle tissue, paving the way to possible transitions to the cardiac tissue. In the work of Nemati et al. (2017) HUVECs were encapsulated in a mixture of gelatin and alginate by the means of an electrostatic encapsulation method at high voltage rate (8kV) followed by extrusion through a 26G needle into a crosslinking solution of CaCl_2_. The composition of the microcapsules allowed cell adhesion and migration through the porous structures; furthermore, the authors observed, *in vitro*, an increased production of NO combined with a high rate of cell survival and an increased tubulogenic activity of encapsulated HUVECs compared to control cells. The superior angiogenic capability of encapsulated HUVECs was also confirmed *in vivo* by injecting cell-laden microcapsules in the muscular tissue of an immune-compromised mouse model. Whereas the direct injection of HUVECs determined an extensive cell spread resulting in the failure of microvascular formation, encapsulated cells promoted an increase of vascular density constituted of both large- and micro-size vascular structures with normal ECs alignment and epithelialization in the periphery. Bushkalova et al. (2019), instead, developed macroporous scaffolds made of alginate-chitosan polyelectrolyte complexes to deliver MSCs in soft tissues. This strategy exploits the association of alginate with a cationic polymer, chitosan, to form a polyelectrolyte complex of opposite charge that can be subsequently freeze-dried and crosslinked; in this way, chemically unaltered polymers preserve their initial biocompatibility while their rheological properties improve and adapt to the mechanical resistance of soft tissues. Instead of pursuing MSCs differentiation into a specific cell type within the scaffold, the main goal of this work was to ensure cells survival, retention, and paracrine activity in order to stimulate the vascularization. *In vitro* tests assessed cell viability and growth, besides an enhanced tendency to secrete FGF2. The most promising results concern the *in vivo* model, implantation in the pectoral muscle of healthy rats, where the team found a good engraftment of the scaffold in the target tissue within 28 days; the progressive degradation of the scaffold was followed by the increasing formation of small functional blood vessels and the reduction of the fibrous capsule. From these studies, it emerged the real possibility of treating MI through the combination of growth factors and a suitable cell source to promote angiogenesis. Undoubtedly, further work is needed to determine which cell source may be suitable in clinical applications, in order to avoid immune-response or off-target vascularization with consequent severe side effects (Epstein et al., 2001).

### Alginate in Cell Delivery Systems

After supporting vascularization of the infarcted area, the second step is to repopulate the damaged area with cells. Mainly, two different cell populations can be implanted, stem cells that may form teratomas, or mature cardiomyocytes, which can cause an electromechanical mismatch within the heart (Chen et al., 2009; Liu et al., 2013). Recently, stem cell-based therapies emerged as possible candidates in treating MI. Unless many studies have been done, at present, only pluripotent stem cells (PCSc) have shown clear ability to differentiate into functional cardiomyocytes (Choe et al., 2019a). Furthermore, cells retention in the heart is very low (Sanganalmath and Bolli, 2013; Hirt et al., 2014); ischemic myocardium displays a hostile environment for cells due to high level of oxidative stress, poor nutrient supply and it offers an open way for host immune system attack. Alginate hydrogels, porous scaffolds, or alginate cell sheets/patches were enrolled to improve cell retention, survival, and function in the ischemic heart. Despite the continuous effort in developing less invasive procedures, the evidence that alternative administration strategies, such as intracoronary injection, may lead to a compromised cell retention (Follin et al., 2018) drove most of the research toward *in situ* application of the developed devices. Partially oxidized alginate was used in preparing an ASDC-loaded conductive H2S-releasing hydrogel system able to improve performances of the infarcted zone in rats (Liang et al., 2019). Specifically, alginate was oxidized through sodium iodate, and tetraaniline (TA) and 2-aminopyridine-5-thiocarboxamide (APTC) were employed to synthetize the multifunctional co-polymer; while TA guaranteed conductive properties, APTC was responsible for inflammatory response inhibition and angiogenic stimuli. Moreover, in a recent *in vitro* study, alginate functionalized with RGD sequence was employed to assess encapsulated cells resistance to oxidative stress. The immobilization of the RGD peptide to sodium alginate was performed utilizing the aqueous carbodiimide chemistry (Shachar et al., 2011), thus the peptide was conjugated to alginate via an amide bond between its terminal amine and the carboxylate on alginate. The results showed that human mesenchymal stem cells (hMSCs) encapsulated in raw alginate or in alginate enriched with RGD have higher resistance to hydrogen peroxide-induced cell death (Choe et al., 2019b). Partially oxidized alginate was also employed in a recent work where PSCs pre-differentiated into the early cardiac lineage were encapsulated into an alginate-chitosan micromatrix (ACM); to create the so-call micromatrix the authors took advantage of the electrostatic interactions among the positively charged chitosan and the negative charges of cell membrane and alginate, by successively soaking cell aggregates in the saline solution of the polymers. These aggregates were injected into hearts of mice showing an increase in cardiac function and survival of animals after MI (Zhao S. et al., 2016). In addition to the previous challenges, another point needs to be addressed, timing of stem cells injection; it was showed that injection of SCs after 4–7 days from the MI improves cells survival but, at the same time, significant injury is accumulated in the ischemic heart (Suzuki et al., 2004; van den Akker et al., 2013). Unless many steps should be done, alginate cell-based therapy seems to be a promising way in the treatment of MI.

### Alginate in Restoring Electrical Conductivity

The last aspect to be taken into consideration about restoring the infarcted area is electrical conductivity. Alteration of the normal cardiac conductive system, resulting in the interruption of connectivity between ion channels and connexins (Huang et al., 2017), is another evidence occurring after MI. CMs death and scar tissue formation play a pivotal role in the conductive connection disruption (Ongstad and Gourdie, 2016). The development of a conductive injectable hydrogel can help transmission of the electrical impulses and maintaining of a stable cardiac rhythm, restoring the normal interchange between the synchronous contractions and the relaxation time (Guo and Ma, 2018). On the contrary, injection of non-electroactive hydrogels, results in an electrically isolated system (Shin et al., 2013), impeding the transmission of electrical impulses to all the infarcted tissue and thus reducing the percentage of success of the regenerative process. Natural hydrogels represent convenient starting substrate to obtain electroactive biomaterials; in particular, thanks to high water level retention, good biocompatibility and their structural resemblance to ECM that enables the diffusion of small molecules (Shi et al., 2016). Moreover, hydrogels can be loaded with conductive components, e.g., nanoparticles (NPs) such as gold nanoparticles (AuNPs), carbon nanotubes (CNT), oligomers or conjugated with conductive polymers, such as polypyrrole (PPy), polythiophene, poly(3,4-ethylendioxythiophene (PEDOT), and Polyaniline (PANi) (Shi et al., 2013; Huang et al., 2017). The main ways to obtain conductive materials are, crosslinking between two or more different materials (Zhang et al., 2014), chemical oxidative *in situ* polymerization with an oxidant to start the reaction (Shi et al., 2012), electrochemical polymerization where hydrogels are used as conducting electrodes (Shi et al., 2014), and post-polymerization/coating by two subsequently hydrogel immersions, the first into monomer solution of conductive polymer and, the second, into an oxidant solution (Wu et al., 2015; Yang et al., 2016; Distler and Boccaccini, 2019; Jiang et al., 2019). Dvir et al. (2011) modified the conductive properties of alginate through a calcium crosslinking strategy incorporating gold nanowires (Alg–AuNW) obtaining a patch where they seeded neonatal rat cardiomyocytes and measured electrical conductivity. In particular, they demonstrated an increased electrical conductivity of the material itself and an increase of electrical conductivity of seeded rat cardiomyocytes. Furthermore, they showed an improvement in cell organization and contraction when the length of gold nanowires surpass the thickness of alginate’s pores (Dvir et al., 2011). Kaklamani et al. (2014) demonstrated that alginate crosslinked with Ca^2+^ is more conductive than alginate crosslinked with Sr^2+^; Sr^2+^ displays a larger ionic radius compare to Ca^2+^, thus, it forms less ionic bridges with alginate hydrogel compared to Ca^2+^. For instance, a higher ions level is associated with larger charge density of the hydrogel (Kaklamani et al., 2014). Alginate has also been used for neural differentiation. The conductive hydrogel composed with polypyrrole and alginate (PPy/Alg) has ten times more electrical conductance compared to solely alginate hydrogel (Yang et al., 2016; DeVolder et al., 2017; Ketabat et al., 2017; Ren et al., 2019). The hybrid polymer is obtained by chemically polymerizing PPy within ionically crosslinked alginate: pyrrole monomers are allowed to diffuse into alginate hydrogels before starting their polymerization with a chemical oxidant (FeCl_3_); further stabilization is provided by the interactions among positively charged pyrrole oligomers and negatively charged alginate. PPy/Alg hydrogel promotes hMSC toward a neural differentiation, implying a possible use for neural tissue engineering (Yang et al., 2016). Interesting would be the evaluation of the same PPy/Alg hydrogel in the contest of cardiomyogenic maturity, or cardiovascular regeneration therapy as well, since the conjugation of these two materials provide an enhanced electrical conductivity, extremely necessary for the cardiac regeneration.

Although great efforts have been put into developing a conductive alginate, suitable to restore normal electrical conductivity in infarcted heart, the way to reach this goal is still very far. In order to restore a normal electromechanical homeostasis within the myocardium the perfect material should be capable to transmit electrical impulses with a proper conduction velocity. Only recently, it emerged the importance to study also the conductivity of injectable hydrogels for cardiac regeneration. To conceive an ideal injectable alginate hydrogel several strategies of manufacture should be combined together. Only the implementation of alginate with cells, vascularization factors and conductive elements would give a “complete material” that can be used for cardiac therapy. The synergy of these three players, together, might be the most successful treatment in heart healing.

## Clinical Trials Implicating Alginate in Cardiovascular Diseases

Food and Drugs Administration (FDA) approved alginate as GRAS in food, pharma, and medicine (wound, bone) applications already in 1970 (Frey et al., 2014; Lee et al., 2015). To date, there are four clinical trials implying alginate in treating heart failure; three of them are completed and the forth one is still recruiting. Algisyl-LVR^TM^ is the most used and commercially available alginate hydrogel implied in sustaining the gradual remodeling of the ventricle in patients subjected to dilated left ventricle (LV), HF, and MI (Lee et al., 2013, 2015; Rao et al., 2016). Until now, two clinical trials have been reported implying Algisyl-LVR^TM^ in restoring infarcted myocardium. Algisyl-LVR^TM^ is conjugated with calcium and sodium (furnished respectively in an aqueous solution and insoluble particles in a 4.6% mannitol solution), reaching a final strength of 3–5 kPa (Lee et al., 2015). Algisyl-LVR is administered during a CABG procedure under general anesthesia. After the intramyocardial injection, it changes the status from liquid to gel remaining permanently in the human heart. Algisyl-LVR^TM^ showed no immune system reactions or rejections, and, thanks to the ability to reduce cardiac tension and cell stress, it enables the natural healing of the heart blocking the disease progression.

## SYM-08-001

The first clinical trial (NCT00847964) was published in 2015, in which Algisyl-LVR^TM^ was administered to 11 patients with dilated cardiomyopathy underwent open-heart surgery with the aim of left ventricular wall augmentation. Only nine patients were able to complete the follow-up at day 8 and 3, 6, 12, 18, and 24 months later, showing promising results. Amelioration of LV size has been observed starting from the 3rd day after the injection procedure. At the last time point of the follow-up (24 months) no deaths occurred and the LV displayed a more ellipsoidal shape with a reduction in the LV end-systolic and LV end-diastolic dimension (LVESD–LVEDD) of respectively 9 and 12%. The ejection fraction (EF) improves of 28% over 24 months while the myofiber stress at the end-of-diastole/systole decreased for 35% over the same period. According to the New York Heart Association (NYHA) classification, the patients’ life quality improved showing a change to a less critical category for almost all patients (II or III class) (Lee et al., 2015). Based on these results, Algisyl-LVR^TM^ has been considered feasible and safe for further clinical trials.

### AUGMENT-HF and AUGMENT-HF II

The second clinical trial performed using Algisyl-LVR^TM^ was AUGMENT-HF (N: NCT01311791). The study was conducted in five different countries with a 1-year-follow-up, where Algisyl-LVR^TM^ was administered to 40 patients (plus 38 control patients for a total number of 78 subjects) aged between 18 and 79 years to modulate HF symptoms and improve heart functions (Anker et al., 2015). After 6 months from injections, the patients showed a progressive increase of the mean peak VO2 (the maximal quantity of O2 in blood that the heart is able to provide to muscles during a physical activity), while the six-minute walk test (6MWT) increased above 300 m threshold for the treated group but decreased in the control group. The 1-year-follow-up demonstrated further enhancement both in mean peak VO2 and in 6MWT in treated group compared to control group, where both conditions slightly worsen with time. These results are correlated with a return to the physical activity for the treated patients and the scaling up the NYHA functional class (I or II) (Mann et al., 2016). An increase in LVEF (16%) was observed after 12 months in the treated group, while LVEDD and left ventricle mass (LVM) resulted decreased for a total of 4 and 9%, respectively (none of the parameters are statistically significant but these findings are unexpected) (Mann et al., 2016). It is possible to deduce that the treatment for HF patients following the standard procedure with the additional Algisyl-LVR injections is a functional strategy to treat HF patients. This polymer lasts permanently in the heart bringing positive effects to the cardiac health (LV augmentation and reduction of LV stress) ameliorating the general mechanical support and life quality of all the treated subjects. To date another clinical trial is ongoing but not yet recruiting, AUGMENT-HF II (NCT03082508). It consists of evaluating the efficacy and safety of Algisyl in LV-augmentation and restoration in patients with dilated cardiomyopathy.

### PRESERVATION-1

Frey et al. (2014) administered in the pivotal study NCT01226563 the so-called hydrogel IK-5001 (BioLineRx, Jerusalem, Israel) to 27 patients (aged between 18 and 75 years) trying to prevent or reverse left ventricular remodeling after a considerable MI (severe-acute) within 7 days from the attack. No control group is present in the study. IK-5001, an implantable bioabsorbable cardiac matrix (BCM) device, is made of 1% sodium alginate plus 0.3% calcium gluconate. IK-5001 hydrogel enters both in the heart and in the extracellular space. Subsequently, it crosslinks in a gel solution thanks to high ionized calcium concentration (characteristic of infarcted myocardium), creating a cardiac scaffold resembling ECM that is bioabsorbable by the tissue and is discarded by kidneys in 3–6 months after the injection (Rao et al., 2015, 2016). In parallel, in the animal model was shown that BCM reabsorption was followed and substituted by a network of capillaries, myofibroblasts and collagen fibrils (Sabbah et al., 2013; Ruvinov and Cohen, 2016). The subjects were controlled after 30, 90, and 180 days from the injections. No further myocardial damages, arrhythmias were observed (Frey et al., 2014). LV remodeling appeared mild recovered with a conservation of LV end-diastolic/end-systolic volume index (LVEDVI – LVESVI) and LVEF parameters. No adverse or serious events have been proven to be correlated with the injections; there was only one episode of syncope after 172 days of administration correlated with the treatment. The whole procedure was well tolerated by patients; in addition, the injections were performed under local anesthesia *via* percutaneous radial artery access and not undergoing an open-heart surgery under general anesthesia (Frey et al., 2014). This pilot study opened the window on a consecutive bigger clinical trial called PRESERVATION-1 (nr: NCT01226563) with the aim to prevent, after one severe MI, ventricle remodeling and heart failure congestion (congestive HF). Two hundred and one subjects were treated with IK-5001 and were compared with 102 healthy subjects. After 6 months, no significant results were obtained. LVEDVI and adverse events remained constant and similar between the two groups. No significant differences have been detected in the improvement of the 6MWT for the treated group; however, a positive and beneficial trend has been shown in the treated group. Severe adverse effects appear to be more frequent in treated subjects (5%) compared to control group (2.9%), on the contrary low rate of mortality and MI have been detected in the treated group compared to the control group. Given these results, neither impressive anatomical changes, nor progress of life quality has been demonstrated in MI subjects. It can be assumed that some results might be linked to the severe MI symptoms that affected some patients that, due to the severity of the damages, could not be treated (Rao et al., 2016).

So far, clinical trials demonstrated the feasibility and safety of treating MI with alginate. Nevertheless, is still necessary to identify the most suitable administration route to exert a therapeutic effect. Indeed, whereas the IK-5001 strategy has the appealing of providing a less-invasive administration, the lack of significant results and life-quality improvements in PRESERVATION-1 trial may indicate a poor specificity for the target area. The direct injection of Algisyl-LVR^TM^ prevents this issue but at the same time underlines the passive role of the biomaterial, whose therapeutic effects seems to rely on providing physical support to the weakened area. Table 2 summarized the discussed clinical trials.

**TABLE 2:** Clinical trials using alginate as basic compound.

**ID number**	**Trial name**	**Status**	**Years**	**Device**	**Condition or disease**	**Participants**	**Description**
NCT00847964	SYM-08-001	Completed	February 2009–November 2012	Algisyl-LVR	Dilated cardiomyopathy	11	A pilot study to evaluate the safety and feasibility of Algisyl-LVR as a method of left ventricular restoration in patients with dilated cardiomyopathy undergoing open-heart surgery (Lee et al., 2015).
NCT01311791	AUGMENT-HF	Completed	August 2012–May 2016	Algisyl-LVR	Heart failure; dilated cardiomyopathy.	78	Randomized, controlled study to evaluate the safety and cardiovascular effects of Algisyl-LVR as a method of left ventricular augmentation in patients with dilated cardiomyopathy (Anker et al., 2015; Mann et al., 2016).
NCT03082508	AUGMENT -HFII	Not yet recruiting	Estimated August 2017–January 2024	Algisyl	Heart failure; dilated cardiomyopathy; heart failure with reduced ejection fraction.	Estimated 240	A pivotal trial to establish the efficacy and safety of Algisyl-LVR in patients with moderate to severe heart failure^1^.
NCT01226563	PRESERVATION-1	Completed	April 2012–December 2015	IK-5001	Acute myocardial infarction; congestive heart failure; ST-elevation myocardial infarction.	303	A placebo controlled, multicenter, randomized double blind trial to evaluate the safety and effectiveness of IK-5001 for remodeling prevention of the ventricle and congestive heart failure after acute myocardial infarction (Frey et al., 2014; Rao et al., 2016).

*^1^https://clinicaltrials.gov/ct2/show/NCT03082508*

## Conclusion

Cardiovascular diseases are the number one cause of death globally. In particular, acute MI, according to WHO, causes 7.3 million deaths worldwide. Despite great strides have been done in the last decade prognosis is still unfavorable, and the search to find new and innovative treatments to heal the infarcted heart is still open. Recently, cardiac regeneration emerged as a promising solution to restore injured heart. The heart is a complex, conductive, and electromechanical organ, thus the necessity to find a durable and conductive material to resist and persist in the “harsh infarcted heart environment.” To date, there are no hydrogels able to mimic cardiac tissue. However, alginate, among all the biomaterials available, seems to be a good candidate for cardiac regeneration. On one hand alginate is a natural polymer, biodegradable, biocompatible, and already approved by FDA for human purposes; moreover, it can be easily modified, it is versatile, and it has affordable production costs. On the other hand, alginate contains many impurities and it is highly hydrophilic. Available results from clinical trials in patients showed that alginate only mildly ameliorates some cardiac parameters but seems to improve patient’s quality of life. To be noted that, until now, all these trials have been focused on the injection of pristine alginate in the damaged area. Despite promising results, so far, no experimentation provided a decisive outcome. Although data from literature show encouraging results, improving vascularization and cell retention in the infarcted area, in a clinical context their potential is limited to temporary solutions and does not show deep ameliorates. To date, all clinical trials imply alginate as mechanical support of the infarcted area, thus the possible premature degradation of alginate inside the organism, in parallel with the incapability of the resident cardiomyocytes to regenerate the infarcted area and the failure in restoring a proper cardiac conductivity, could be the reasons of the partial outcome of the clinical trials. In order to overcome this issue could be useful to complete the stage of cellularization of the alginate matrix *in vitro* with cardiomyocytes; nevertheless, it is well known the difficulty of handling mature cardiomyocytes *in vitro* and the ethical limitations. Moreover, particular attention should be paid to electrical conductivity to prevent arrhythmias, both inserting an alginate matrix capable of substitute the infarcted area and transmit the electrical signal or guiding the cardiomyocytes within the scaffold avoiding spontaneous beating activity. Furthermore, it is necessary to know better the mechanisms underneath the remodeling occurring in the infarcted heart in order to establish when, where, and how much material to inject. So far, the use of alginate in cardiac regeneration seems to be promising, even though a deeper characterization is needed for the eventual translation to the clinical field.

## Author Contributions

GC, AG, AR, MM, and CC designed and wrote the manuscript. RF and PP read and commented the manuscript. GC, AG, and CC designed the study and edited the final manuscript with input from all authors.

## Conflict of Interest

The authors declare that the research was conducted in the absence of any commercial or financial relationships that could be construed as a potential conflict of interest.

## Footnotes

1 https://www.who.int/news-room/fact-sheets/detail/noncommunicable-diseases

2 http://wayback.archive-it.org/7993/20171031063235/https://www.fda.gov/Food/IngredientsPackagingLabeling/GRAS/SCOGS/ucm260857.htm

## Abbreviations

ADSCs: adipose derived stem cells
CMs: cardiomyocytes
CVDs: cardiovascular diseases
ECs: endothelial cells
ECM: extracellular matrix
EDD: end-diastolic dimension
EF: ejection fraction
ESD: end-systolic dimension
FDA: Food and Drugs Administration
G: 1,4 α -L-guluronic acid
GRAS: generally regarded as safe
hMSCs: human mesenchymal stem cells
LV: left ventricle
LVM: left ventricle mass
M: 1,4-linked β -D-mannuronic acid
MI: myocardial infarction
mPSCs: mouse pluripotent stem cells
MSCs: mesenchymal stem cells
NCD: non-communicable disease
PSCs: pluripotent stem cells
rMSCs: rat mesenchymal stem cells
VEGF: vascular endothelial growth factor
VSMCs: vascular smooth muscle cells.
